# Therapeutic efficacy of proton transport inhibitors alone or in combination with cisplatin in triple negative and hormone sensitive breast cancer models

**DOI:** 10.1002/cam4.4371

**Published:** 2021-11-18

**Authors:** Enrica Balza, Sebastiano Carlone, Sonia Carta, Patrizia Piccioli, Vanessa Cossu, Cecilia Marini, Gianmario Sambuceti, Anna Rubartelli, Patrizia Castellani

**Affiliations:** ^1^ Cell Biology Unit IRCCS Ospedale Policlinico San Martino Genoa Italy; ^2^ Nuclear Medicine IRCCS Ospedale Policlinico San Martino Genova Italy; ^3^ Department of Health Sciences University of Genoa Genoa Italy; ^4^ Bioimaging and Physiology (IBFM) CNR Institute of Molecular Milan Italy; ^5^ Vita‐Salute San Raffaele University Milan Italy

**Keywords:** breast cancer, cisplatin, combo therapies, immune response, proton exchanger inhibitors

## Abstract

Triple negative breast cancers (TNBCs) are very aggressive and have a poor prognosis due to lack of efficacious therapies. The only effective treatment is chemotherapy that however is frequently hindered by the occurrence of drug resistance. We approached this problem in vitro and in vivo on a triple negative and a hormone sensitive breast cancer cell lines: 4T1 and TS/A. A main defense mechanism of tumors is the extrusion of intracellular protons derived from the metabolic shift to glycolysis, and necessary to maintain an intracellular pH compatible with life. The resulting acidic extracellular milieu bursts the malignant behavior of tumors and impairs chemotherapy. Therefore, we investigated the efficacy of combined therapies that associate cisplatin (Cis) with proton exchanger inhibitors, such as esomeprazole (ESO) and 5‐(*N*‐ethyl‐*N*‐isopropyl)amiloride (EIPA). Our results demonstrate that in the 4T1 triple negative model the combined therapy Cis plus EIPA is significantly more effective than the other treatments. Instead, in the TS/A tumor the best therapeutic result is obtained with ESO alone. Remarkably, in both 4T1 and TS/A tumors these treatments correlate with increase of CD8^+ ^T lymphocytes and dendritic cells, and a dramatic reduction of M2 macrophages and other suppressor myeloid cells (MDSC) in the tumor infiltrates.

## Graphical abstract

We show that association of cisplatin and sodium/hydrogen exchanger one inhibitors decreases tumor growth, improves the tumor microenvironment state, and increases survival more efficiently than cisplatin alone in triple negative breast cancer‐bearing mice, supporting the exploitation of this combo therapy in chemoresistant TNBCs. At variance, esomeprazole alone is more efficacious than cisplatin or various combo therapies in hormone sensitive breast cancer in mice. Thus, proton transport inhibitors should be considered as valuable nontoxic anticancer drugs.

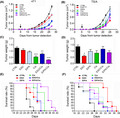

## 1 INTRODUCTION

Breast cancer is one of the most common causes of women mortality. The majority of breast cancers express receptors for estrogens and/or progesterone (~75%), while HER2 is upregulated in ~20% of the cases. About 15% of breast cancers are triple negative (TNBC), meaning that they lack hormone receptors and overexpression of HER2. These cancers are highly aggressive with poor prognosis and lack targeted therapies.^1^, ^2^ Chemotherapy is currently the only option of treatment, and several classes of drugs, including platinum agents, have been exploited to treat TNBC.^3^ Although an increase in life span is often achieved, the therapeutic value of these drugs is low, also due to the frequent development of drug resistance.^4^

Based on these observations, there is intense interest in finding new medications that can cure TNBCs and other aggressive cancers. Anticancer combo therapies include the association of different chemotherapeutics, or of one chemotherapy drug (or radiotherapy) with biologics.^5^ Combo therapies using various chemotherapeutics may increase anticancer efficacy while reducing the optimal dose of each drugs, thus decreasing adverse effects.^6^ The simultaneous use of more than one agent also minimizes the chance of relapse unless mutations conferring resistance to different drugs arise.

An interesting, unconventional combo therapy to treat cancer is the association of a chemotherapeutic agent with nontoxic drugs targeting tumor defenses.^7^, ^8^ A major defense mechanism is evolved in tumor cells to eliminate lactate and other acidic metabolites caused by the Warburg effect, a metabolic phenomenon characterized by increased glucose uptake and fermentation resulting in increased cell proliferation.^9^, ^10^ A decrease in intracellular acidic catabolites is possible thanks to the upregulation (or relocalization) of enzymes^11^ and/or transporters, including v‐ATPase^12^ and NHE‐1,^13^ and leads to a decrease in extracellular pH (pHe) linked to an increase in intracellular pH (pHi).^13^, ^14^ Swapping pHi and pHe has a double advantage for cancer cells: the pHi becomes compatible with life, whereas the acidic pHe facilitates tumor progression in various ways. Among these, the low pHe alters the capacity of chemotherapeutics including cisplatin, doxorubicin, paclitaxel to enter cells^14^, ^15^ and consequently induces drug resistance.^16^, ^17^ A different mechanism of drug resistance due to pH swapping, proposed for cisplatin in melanoma, involves sequestration of the drug in the extracellular compartment and its elimination from tumor cells through exosomes.^18^

Consistently, modulation of pH in tumors during chemotherapies was found to increase sensitivity to chemotherapeutic agents.^19^ v‐ATPases are normally restricted to intracellular acidic organelles, but translocate to the plasma membrane in tumor cells.^20^, ^21^ A seminal study by Luciani et al., showed that pretreatment with PPI drugs, largely used to treat gastric acidic hypersecretion, resulted in strong improvement of retention of cytotoxic agents into the cytoplasm of neoplastic cells.^22^ Later, PPIs were shown to reverse chemoresistance by inhibiting v‐ATPase in a model of gastric cancer.^23^ Remarkably, a recent pilot clinical trial showed that intermittent high‐dose PPI enhanced the antitumor effects of chemotherapy in metastatic breast cancer patients without evidence of additional toxicity.^24^ Furthermore, PPI exerted antitumor effects even without association with chemotherapy.^25^ We recently showed that the PPI esomeprazole (ESO) decreases sarcoma and melanoma cell growth and migration by restoring a physiologic pH.^8^

The Na^+^/H^+^ exchanger 1 (NHE‐1) is also aberrantly elevated in tumors displaying a switch between pHe and pHi values and is responsible for drug resistance.^14^, ^26^ Interestingly, NHE‐1 expression was found to be involved in the pathogenesis of TNBC.^14^ Drugs that inhibit NHE‐1, such as cariporide and amiloride, approved for therapy of hypertension and edema following heart failure, were proposed to reverse pH alkalinization and transformed phenotype in human myeloma.^27^, ^28^ Remarkably, the amiloride derivative 5‐(*N*‐ethyl‐*N*‐isopropyl)amiloride (EIPA), 200 times stronger than amiloride in blocking the NHE‐1 antiporter,^29^ sensitizes tumor cells to chemotherapeutic drugs increasing their intracellular accumulation and effectiveness.^30^, ^31^, ^32^

In this study, we used 4T1 triple negative breast cancer and TS/A hormone sensitive breast cancer, in vitro and in vivo in syngeneic mice, to investigate whether the association of Cis to ESO or EIPA is advantageous over use of Cis alone. Our results indicate that while in the hormone sensitive TS/A tumors the best therapeutic effect was provided by ESO alone, in the 4T1 TNBC model the combined treatment Cis plus EIPA was more effective than the other treatments. Remarkably, these effects correlate with increase in CD8^+^ T cells, DCs, tumor‐associated macrophages (TAM)‐M1, and with dramatic reduction in TAM‐M2 and MDSC within the tumor infiltrate.

## 2 MATERIALS AND METHODS

### 2.1 Reagents

The following reagents and antibodies were used: ESO, EIPA, Crystal violet, (Sigma‐Aldrich); LysoSensor Green DND‐189, (Thermo Fisher Scientific); Cis (Accord Healthcare); rabbit anti‐v‐ATPase (TCIRG1, Proteintech); rabbit anti‐NHE‐1 and rat anti‐mouse CD11b (Novus Biologicals); rat anti‐mouse CD206 (AbD Serotec); rat anti‐mouse CD86 clone PO.3 (Millipore); rat anti‐mouse CD4, CD8, CD205 (DEC205) and Ly‐6G/Ly6C (Gr‐1) (Biolegend); rat anti‐mouse CD11b (Novus Biologicals); and rat anti‐mouse CD31(clone MEC 13.3) kindly supplied by A. Mantovani.

### 2.2 Tumor cell lines and culture

The murine breast carcinoma cell lines TS/A (RRID:CVCL_F736) (hormone sensitive breast cancer) kindly provided by Prof. R. Accolla (University of Insubria) was generated as reported.^33^ 4T1 (RRID:CVCL_0125) (triple negative breast cancer‐TNBC) was purchased from ATCC (American Type Culture Collection). Cell lines were routinely tested for mycoplasma contamination using MycoAlert Mycoplasma Detection Kit (Lonza Walkersville Inc.).

### 2.3 Measurement of intracellular pH change

4T1 and TS/A cell lines untreated or treated for 24 h with EIPA and ESO, respectively, were stained with 1 μM LysoSensor Green DND‐189 (30 min at 37°C). Images were analyzed by confocal microscopy as described.^8^

### 2.4 Determination of cell survival

Cell viability was determined as described.^7^ Dose–response experiments (Figure S1) have identified the following concentrations: ESO 100 μM, EIPA: 10 μM, and Cis: 2 μM, respectively. The effects of drugs alone or in combination on cell survival were determined on cells cultured at pH 7.4 or 6.5 as described.^34^ After various time points from culture at pH 7.4 or pH 6.5, the percent of survival of treated cells was calculated versus the specific control (untreated cells at pH 7.4 or pH 6.5).

### 2.5 Animal tumor models

Eight‐ to 10‐week‐old BALB/c mice (Envigo) were subcutaneously implanted with 4T1 (0.1 × 10^6^) and TS/A (0.3 × 10^6^) murine cell lines. Tumor volume was determined and euthanasia was performed as described.^7^, ^8^

### 2.6 Protocols of in vivo treatments

When the tumors reached a volume of 0.15 cm^3^, groups of eight tumor‐bearing mice received the therapeutic treatments with ESO, EIPA, and Cis alone or Cis plus ESO and Cis plus EIPA. Schedule of treatments: ESO 12.5 mg/kg/200 μl saline (ip) three times/week; EIPA 2.5 mg/kg/200 μl saline (ip) daily; and Cis 5 mg/kg/200 μl saline (ip) once a week for 2 weeks. In the combined treatments, the drugs were administered at least 6 h after each other. The welfare of the animals was checked daily and weight loss never exceeded 10% during the treatments.

### 2.7 Staining procedures and immunohistochemistry

Serial cryostat sections of 4T1 and TS/A tumors were processed for immunohistochemistry as described.^35^ Images were acquired and analyzed as described.^8^

For immunofluorescence, 6‐µm‐thick serial cryostat sections from mice tumor samples were fixed with cold acetone for 10 min and double‐stained with the following Abs: rat anti‐mouse mAb to CD206 and rabbit anti‐v‐ATPase or rat anti‐mouse mAb to CD206 and rabbit anti‐NHE‐1. The secondary antibodies used were Alexa Fluor 546 and Alexa Fluor 488 conjugated. Images were analyzed by confocal microscopy and the fluorescence was quantified using ImageJ software.

### 2.8 Statistical analysis

All results were analyzed for statistical significance by *t*‐test or one‐way ANOVA with Bonferroni post‐test by GraphPad Prism (version 4.0). The Kaplan–Meier analysis compared by the Mantel–Cox test was used for survival rate. All error bars represent SEM. *p*‐values ≤0.05 were considered significant.

## 3 RESULTS

### 3.1 Both NHE‐1 and v‐ATPase are expressed by 4T1 and TS/A murine mammary cancer cells.

**FIGURE 1:**
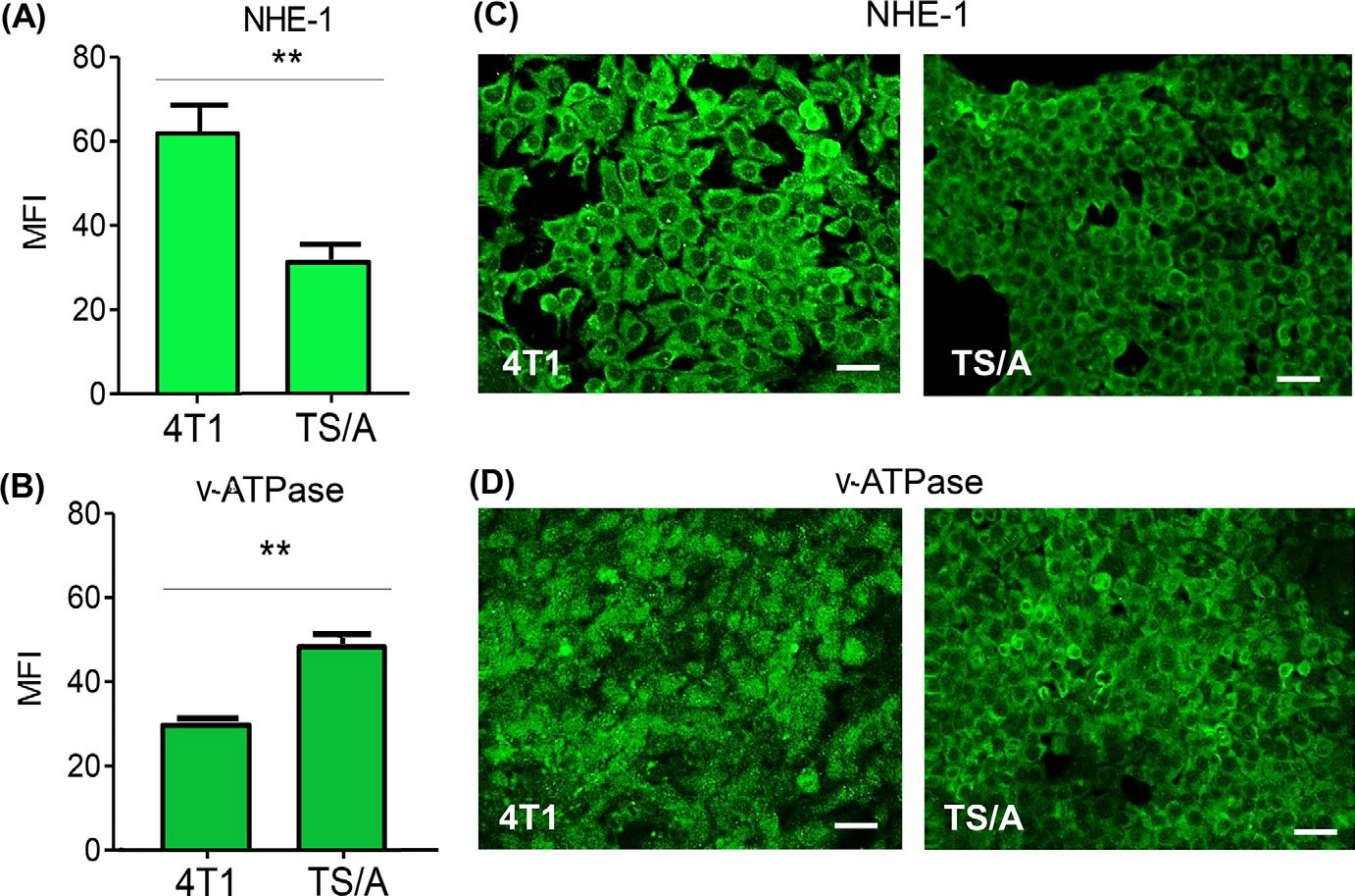
NHE‐1 and v‐ATPase expression on 4T1 and TS/A breast cancer cells. Flow cytometry analysis (A, B) and immunofluorescence representative images (C, D) of 4T1 and TS/A cell lines stained with anti‐NHE‐1 (A, C) or anti‐v‐ATPase (B, D) Abs. Scale bar, 30 µm

We investigated the expression of NHE‐1 and v‐ATPase in the TNBC 4T1 and the hormone sensitive TS/A murine breast cancer cell lines. Flow cytometry showed relevant surface expression of NHE‐1 in both cell lines, at a higher extent in 4T1 cells (Figure 1A). In contrast, surface v‐ATPases were more abundant on TS/A than on 4T1 cells (Figure 1B). Confocal analyses confirmed the higher expression of NHE‐1 in 4T1 cells (Figure 1C) and a prevalent intracellular localization of v‐ATPase (Figure 1D), consistent with the physiologic endolysosomal localization of this proton pump.^12^

### 3.2 EIPA and ESO alone or in combination with Cis, inhibit 4T1 and TS/A cell proliferation and increase intracellular acidity.

Next, we exposed 4T1 and TS/A cells for 96 h to the NHE‐1 inhibitor EIPA or to the PPI ESO alone or together, or in combination with Cis, at the doses and times previously selected for the treatment of tumor cell lines (Figures S1 and S2).^8^, ^36^ Since the association of ESO and EIPA did not increase the effects of each proton pump inhibitor alone (data not shown), the combo therapy with ESO/EIPA was excluded.

**FIGURE 2:**
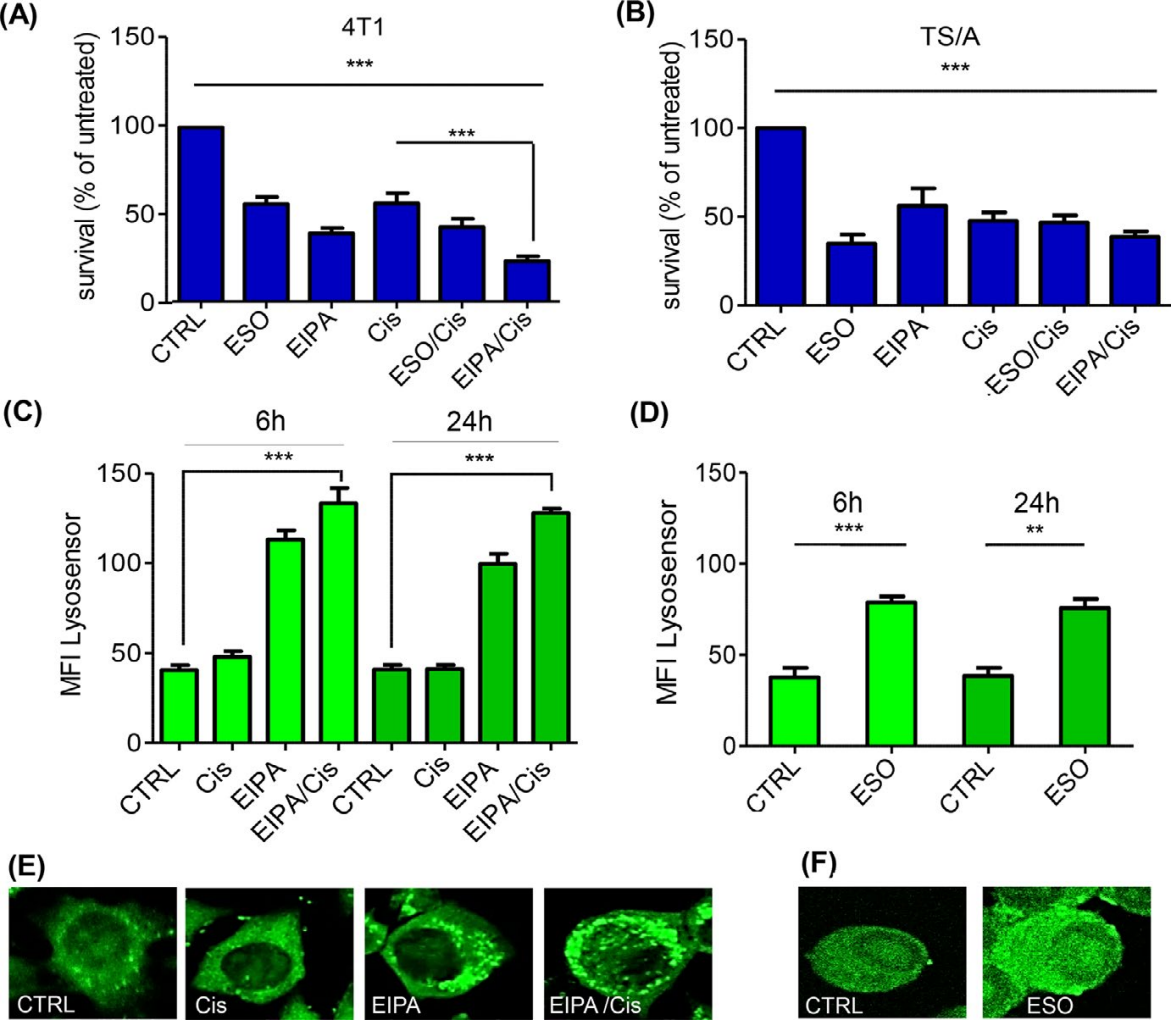
Effect of ESO, EIPA, and Cis on cell growth and pHi in vitro. Survival at 96 h of 4T1 (A) and TS/A (B) cells untreated or treated with ESO, EIPA, and Cis, alone or in combination. Data are expressed as percent of untreated cells (mean of three experiments ± SEM, *** *p *< 0.001). LysoSensor Green DND‐189 positivity in 4T1 cells (C, E), untreated (Ctrl) or treated with Cis, EIPA, and EIPA/Cis as indicated, and in TS/A cells (D, F), Ctrl or treated with ESO. (C, D): Quantification of fluorescence levels in 4T1 (C) and TS/A (D) cells at 6 and 24 h after treatment. Mean fluorescence intensity (MFI) was obtained in 10 fields ± SEM (**p < 0.01, ***p < 0.001). (E, F): Representative images of LysoSensor‐stained cells. Magnification 400x

Culture in the presence of ESO reduced the number of surviving cells in a time‐dependent manner, at a greater extent in TS/A than in 4T1 cells. In contrast, EIPA treatment was more efficacious in 4T1 cells (Figure 2 and Figure S2). In both models, the effects of ESO and EIPA on cell survival were similar, or even greater, than those observed with the chemotherapeutic agent Cis alone (Figure 2A and B). We therefore studied whether the association with EIPA or ESO could enhance the efficacy of Cis treatment. The results (Figure 2A) show that, at 96 h from treatment, the concomitant exposure of 4T1 cells to EIPA and Cis (EIPA/Cis) was more efficacious than either drug alone, with a significant decrease in cell survival (survival rate: 23% with EIPA/Cis vs. 39% and 56% with EIPA and Cis, respectively). Also the combo treatment ESO plus Cis (ESO/Cis) was more efficient than the single treatments (survival rate: 43% with ESO/Cis vs. 56% with ESO or Cis) (Figure 2A), although at a lesser extent than EIPA/Cis. In contrast, in TS/A cells ESO/Cis was not more effective than ESO alone (Figure 2B).

We then tested whether treatments with EIPA/Cis in 4T1 cells and ESO in TS/A cells are associated with changes in intracellular pH. Cells exposed 6 or 24 h to the drugs were stained with LysoSensor and analyzed by confocal microscopy (Figure 2C–F). Remarkably, EIPA or EIPA/Cis‐treated 4T1 cells (Figure 2C,E) and ESO‐treated TS/A cells (Figure 2D,F) displayed increased intracellular acidity, with more LysoSensor‐positive intracellular organelles, of larger size than in untreated cells.

To investigate whether the low pH of tumor microenvironment affects drug efficacy, 4T1 and TS/A cells were incubated either in buffered standard condition (pH 7.4) or low pH (pH 6.5). The results show that while EIPA and Cis, as well as the two drugs together, are more efficient in reducing survival at pH 7.4, the efficacy of ESO alone or associated to Cis is higher at pH 6.5 (Figure S3).

### 3.3 In vivo therapeutic effects of EIPA and ESO alone or associated to Cis.

**FIGURE 3:**
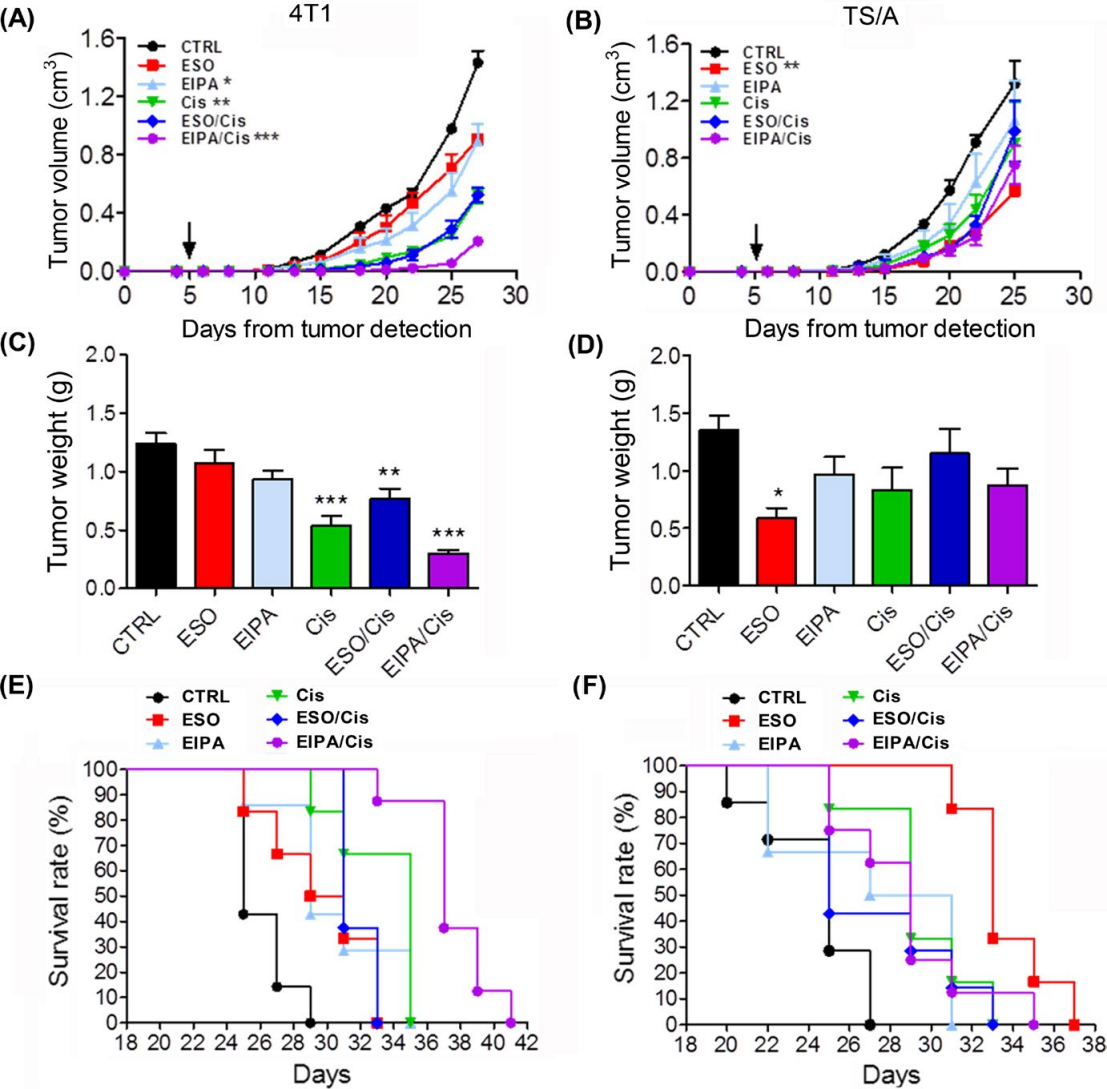
Reduction of tumor growth in vivo in response to the different treatments. Mice injected with 4T1 (A) or TS/A (B) cells were untreated or treated with ESO, EIPA, and Cis alone or in combination. (A, B): Tumor volumes of untreated and treated tumor‐bearing mice were measured throughout the experiment and results are expressed as cm^3^ (mean ± SEM). (C, D): At the end of all therapeutic treatments tumor weights of 4T1 (C) and TS/A (D) were compared (g, mean ± SEM). Data are illustrative of eight mice per each treatment group. *p < 0.05, **p < 0.01, ***p < 0.001 (E, F): Survival was monitored up to 41 days for 4T1 (E) and up to 37 days for TS/A (F). (E) Survival EIPA/Cis versus CTRL, versus EIPA, versus ESO, and versus ESO/Cis: *p *< 0.001; EIPA/Cis versus Cis: *p *< 0.01; (F) Survival ESO versus CTRL: *p *< 0.001; Survival ESO versus EIPA and versus ESO/Cis: *p *< 0.01; Survival ESO versus EIPA/Cis and versus Cis: *p* < 0.05

To test the efficacy of EIPA and ESO in vivo, we carried out experiments of syngeneic transplantation in Balb/c mice. 4T1 and TS/A cells were inoculated in six groups of mice (Figure 3). One group was left untreated, the others were subjected to treatment with Cis, EIPA, and ESO alone and in combination (EIPA/Cis and ESO/Cis). Therapies were started when the tumor became palpable, which in most experiments occurred at day 5 from cell injection. Untreated mice were sacrificed when the tumor volume reached 1.2–1.5 cm^3^. None of the therapies caused pathological alterations or weight loss in tumor‐bearing mice (data not shown). However, all therapies reduced the rate of tumor growth and tumor weight compared to untreated mice (Figure 3A–D). In the 4T1 tumor model, single treatments with EIPA or ESO, and the combo therapy with ESO/Cis were less effective than treatment with Cis alone. In contrast, the combination of EIPA/Cis was the most efficient in delaying tumor growth and reducing tumor weight. At the endpoint, after 22 days from the beginning of the treatments, the tumor size of EIPA/Cis‐treated mice was about 80% smaller than that of untreated mice (Figure 3A,C). The antitumor effect of EIPA/Cis evaluated by *q*‐value^37^ indicated additive effect between the two drugs. In agreement, the survival curves indicate that the best treatment for 4T1 tumors was the combo therapy EIPA/Cis (Figure 3E). EIPA/Cis‐treated mice displayed the longest survival (30% more than untreated mice), with a significantly higher effects on survival comparing to the other drugs, used alone or associated. (Figure 3E).

In the TS/A tumor model, the most effective treatment was ESO alone that induced the strongest inhibition of tumor growth and weight (Figure 3B,D). Consistently, mice treated with ESO alone displayed an overall survival about 27% longer than untreated mice. The other drugs alone or in association exhibited intermediate effects, in all cases significantly lower than ESO alone (Figure 3F).

### 3.4 Modulation of angiogenesis by single or combo therapy.

**FIGURE 4:**
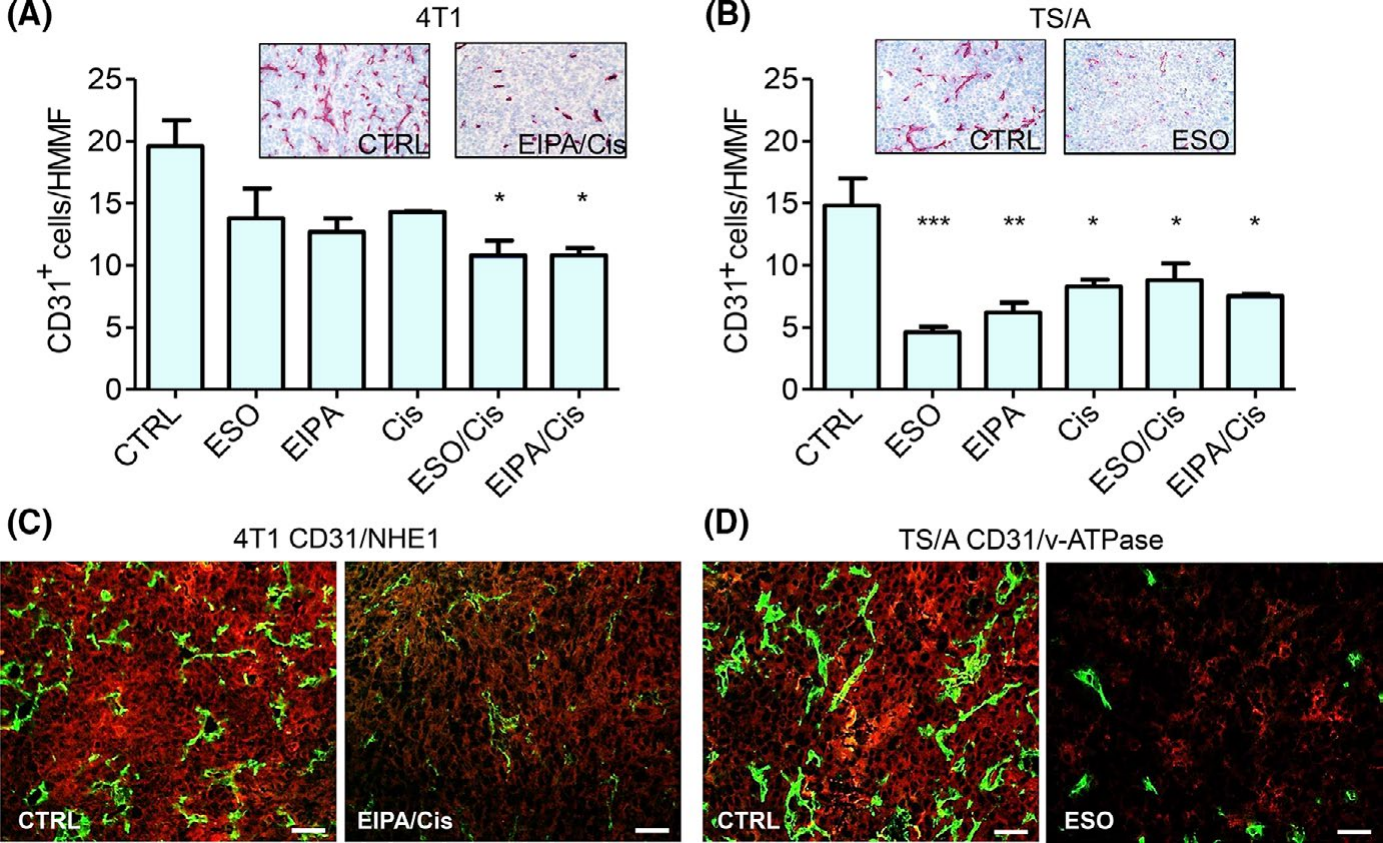
Immunohistochemical assessment of intratumor endothelial cells. (A, B): CD31‐positive cells in untreated (CTRL) or treated 4T1 (A) and TS/A (B) tumor‐bearing mice. Results are expressed as cell number (mean +/− SEM) per high‐magnification microscopic field (HMMF). Data are representative of at least three mice per each treatment group (*p < 0.05, **p < 0.01, ***p < 0.001). Inset A, B: Representative images of immunohistochemical staining with anti‐CD31 Ab of CTRL and EIPA/Cis‐treated 4T1 tumors (A) and of CTRL and ESO‐treated TS/A tumors (B). C) Double immunofluorescence staining with anti‐CD31 (green) and anti‐NHE‐1 (red) Abs of 4T1 tumors from CTR or EIPA/Cis‐treated mice. (D) Double immunofluorescence staining with anti‐CD31 (green) and anti‐v‐ATPase (red) Abs of TS/A tumors from CTR or ESO‐treated mice. Scale bar, 30 μm

Both 4T1 and TS/A tumors from untreated mice displayed a strong vascularization evaluated by CD31 staining that was decreased by the different treatments (Figure 4A,B). In particular, in 4T1 tumors, reduction in vessel density was significant with either combo treatments (ESO/Cis or EIPA/Cis) but little with single treatments (Figure 4A). In contrast, in TS/A tumors all treatments were efficacious in decreasing vascularization, although the best was ESO alone that provided 70% and 50% reduction in vessels with respect to tumors from untreated or Cis‐treated mice, respectively (Figure 4B). No co‐stain of anti‐CD31 with anti‐NHE‐1 or v‐ATPase Abs was observed in 4T1 and TS/A tumors, untreated or treated with EIPA/Cis or ESO, respectively (Figure 4C,D).

### 3.5 Modulation of intratumor immune cells by single or combo therapy.

We then investigated the presence of tumor‐infiltrating immune cells^38^ and their modulation by the various therapies.

**FIGURE 5:**
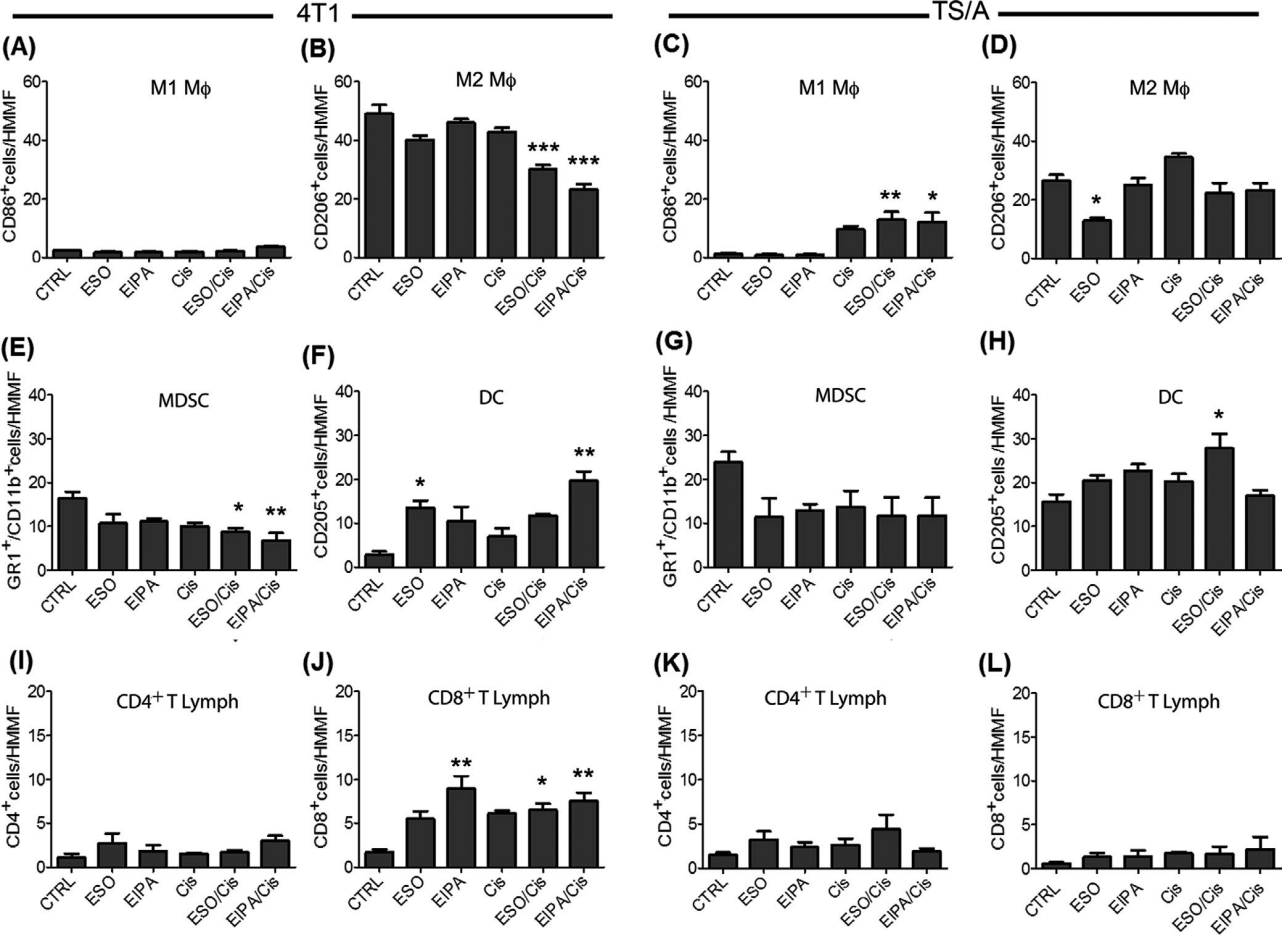
Immunohistochemical assessment of tumor‐infiltrating immune cells. Immunohistochemical assessment in untreated (CTRL) and treated 4T1 (A, B, E, F, I, J) and TS/A (C, D, G, H, K, L) tumor‐bearing mice. CD86^+^M1 macrophages (A, C), CD206^+^ M2 macrophages (B, D), Gr‐1^+^/CD11b^+^ MDSCs (E, G), ^+^DCs (F, H), CD4^+^ T lymphocytes (I, K), and CD8^+^ T lymphocytes (J, L). Results are expressed as cell number per HMMF. Data are representative of at least three mice per each treatment group (mean +/− SEM, *p < 0.05, **p < 0.01, ***p < 0.001)

The number of M1 macrophages was very low in both tumors from untreated mice (Figure 5A,C) and unaffected by all therapies in 4T1 tumors (Figure 5A), while increased by Cis alone and by the two combo therapies in TS/A tumors (Figure 5C). On the contrary, M2 macrophages were highly represented, being more abundant in 4T1 (Figure 5B) than in TS/A untreated tumors (Figure 5D). In 4T1 tumors, both combo therapies decreased M2 macrophages, the most effective treatment being EIPA/Cis (Figure 5B). In TS/A tumors, M2 macrophages showed an important reduction in mice treated with ESO alone (Figure 5D).

A relevant infiltration of MDSCs was also observed in both tumors (Figure 5E,G). Although the number of MDSCs was decreased by all treatments, the strongest reduction was again obtained by EIPA/Cis in 4T1 tumors (Figure 5E) and ESO in TS/A tumors (Figure 5G).

DCs were very low in 4T1 tumors and increased by all treatments especially by EIPA/Cis that increased the number of DC by sevenfold (Figure 5F). In TS/A tumors the basal infiltration of DC was higher, and the drug‐induced increase was overall less strong (Figure 5H).

In both tumors, natural killer (NK) cells (data not shown) and CD4^+^ T cells (Figure 5I,K) were few and poorly modulated by the treatments. Also CD8^+^ T lymphocytes were in low number in both tumors (Figure 5J,L). However, in 4T1 tumors, CD8^+^ T cells were dramatically increased by all treatments, especially by EIPA and EIPA/Cis (Figure 5J).

Together, the data show that the most efficacious therapies, that is, EIPA/Cis in 4T1 and ESO alone in TS/A tumors (Figure 3), not only affect tumor cells but also the tumor microenvironment, with decrease of infiltrating M2 macrophages and MDSC. Furthermore, EIPA/Cis also increased infiltrating DCs and CD8^+^ T cells in 4T1 tumors.

### 3.6 NHE‐1 and v‐ATPases are expressed by M2 macrophages and are modulated by therapies.

**FIGURE 6:**
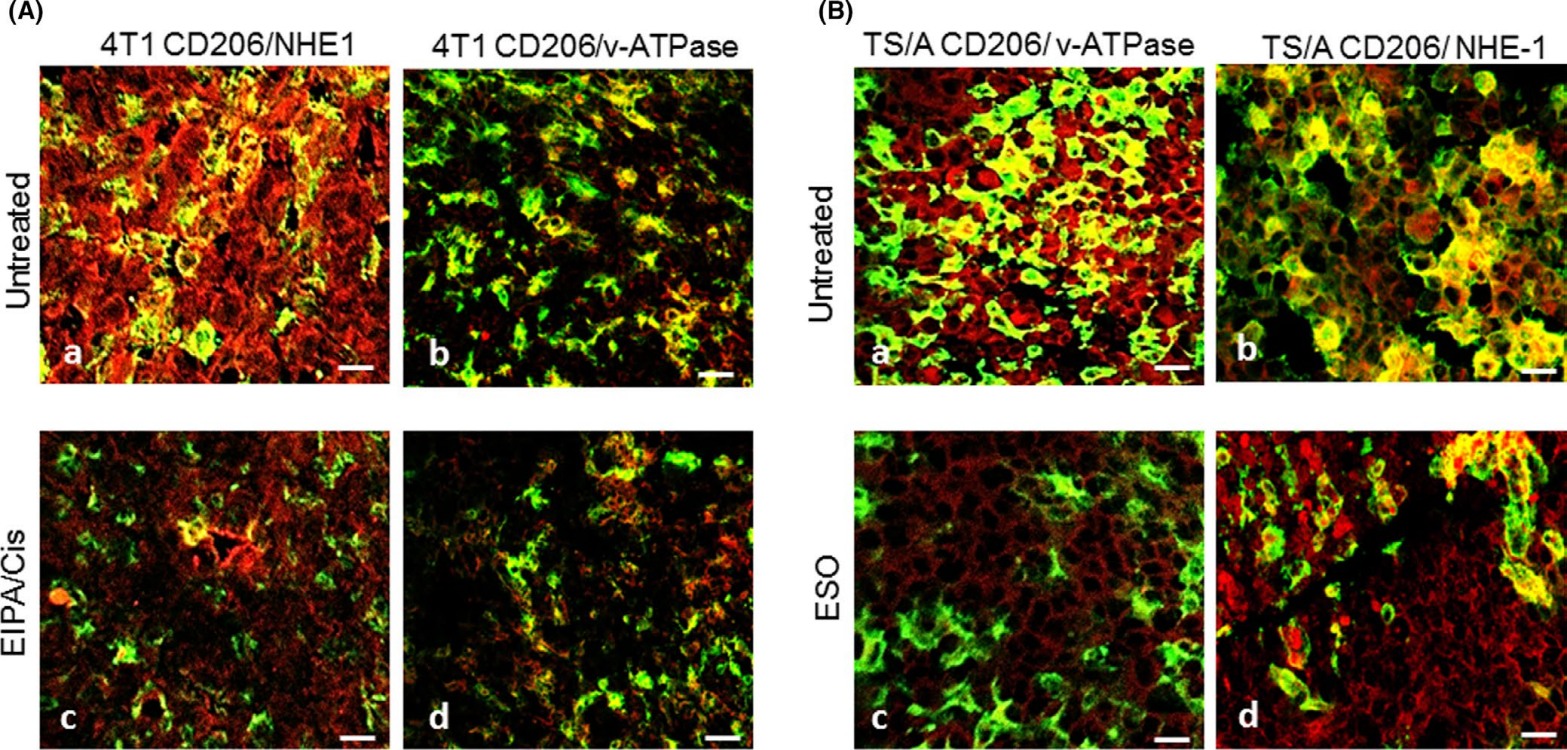
Reduction of intratumor M2 macrophages by EIPA/Cis and ESO treatments. (A) Double immunofluorescence analysis with anti‐CD206 (green) and anti‐NHE‐1 (red, a and c) or anti‐v‐ATPase (red, b and d) of 4T1 tumors from untreated (a and b) or EIPA/Cis‐treated (c and d) mice. (B) Double immunofluorescence analysis with anti‐CD206 (green) and anti‐v‐ATPase (red, a and c) or anti‐NHE‐1 (red, b and d) of TS/A tumors from untreated (a and b) or ESO‐treated (c and d) mice. Scale bar, 30 μm

To assess whether treatments with EIPA/Cis (on 4T1 cells) or ESO (on TSA cells) affect their expression on cancer cells and M2 infiltrating macrophages, tumors sections from untreated or treated mice were co‐stained with CD206 and anti‐NHE‐1 or v‐ATPase Abs. As shown in Figure 6Aa,c untreated 4T1 tumors express high levels of NHE‐1 in both cancer cells and infiltrating M2 macrophages that were strongly reduced in EIPA/Cis‐treated tumors. In contrast, v‐ATPase was very low in tumor cells and moderate in M2 macrophages in tumors from both untreated and EIPA/Cis‐treated mice (Figure 6Ab,d).

Conversely, in TS/A tumors, v‐ATPases were highly expressed both in cancer cells and M2 macrophages and strongly decreased in ESO‐treated tumors (Figure 6Ba,c). NHE‐1 was more expressed by M2 macrophages than by cancer cells in control tumors and almost unaffected in tumors treated with ESO (Figure 6Bb,d).

## 4 DISCUSSION

In this paper, we propose a novel therapeutic approach to breast cancers based on a combo therapy that comprises cisplatin associated to proton transport inhibitors. In particular, the combination of Cis and EIPA strongly decreased the survival of the triple negative 4T1 mammary tumor cells in vitro, and results in a remarkable tumor growth delay, reduced tumor weight, and increased survival in vivo. In contrast, this association did not increase the therapeutic efficacy of Cis alone in the hormone sensitive TS/A mammary cancer. However, in this model, treatment with the proton pump inhibitor ESO alone displayed higher therapeutic value than Cis and combo therapies, both in vitro and in vivo. The different response to EIPA and ESO most likely depends on the different degree of surface expression of the two proton exchangers by the two cell lines. Plasma membrane expression of NHE‐1 by 4T1 cells is higher than by TS/A cells, whereas v‐ATPase is highly expressed on the external membrane of TS/A cells, whereas it is mainly intracellular in 4T1 cells.

The rationale for associating proton transport inhibitors with Cis bases on the evidence that some tumors display resistance to antineoplastic drugs due to the acidic extracellular environment generated by the upregulation of transporters that extrude protons.^9^, ^10^, ^11^ ^,^ ^15^, ^16^, ^17^ Previous in vitro analyses proposed that blocking NHE‐1 and v‐ATPase with amiloride and PPI, respectively, in different tumor models induces a swap of the pH gradient between extracellular environment and tumor cells.^14^, ^19^ Consistently, in our experiments, EIPA increased pHi of 4T1 cells and ESO increased pHi in TS/A cells. ESO, but not EIPA, alone or in combination, increases its antitumor activity at low extracellular pH, in agreement with the notion that ESO is a prodrug, activated at low pH, as also confirmed in studies on tumor cells.^34^

The finding that EIPA increases the effectiveness of Cis in 4T1 tumors both in vitro and in vivo is consistent with the observation that knockout of NHE‐1 in a triple negative human mammary cell line decreased its ability to form xenografts in nude mice, and increased its sensitivity to paclitaxel in vitro.^32^ Our results extend these observations and show that not only EIPA strongly increases the cytotoxic effect of Cis on 4T1 tumor cells, but also specifically reduces tumor‐associated blood vessels and myeloid cells such as M2 macrophages and MDSCs. Both vessels and suppressive myeloid cells are known to participate in cancer progression.^38^, ^39^

NHE‐1 is ubiquitously expressed in mammalian cells, at different extents,^13^ and is modulated by stress.^40^, ^41^ In both 4T1 and TS/A tumor models, not only tumor cells, but also M2 macrophages express significantly NHE‐1, whereas endothelial cells are negative. Therefore, EIPA is likely to act directly on infiltrating myeloid cells that express NHE‐1, but indirectly on vessels cells, whose decrease may be secondary to the effects of the combo treatment on the tumor.

Whereas most non‐transformed cell types express v‐ATPase only intracellularly,^12^ macrophages, may express these transporters also on the plasma membrane.^8^, ^42^ Here, we observed high expression of membrane v‐ATPase on the infiltrating macrophages in both tumor models. Like EIPA/Cis on 4T1, ESO alone in TS/A not only decreases tumor burden but also reduces tumor vascularization and infiltration by M2 macrophages and MDSCs. This result is in line with the reduction of infiltrating M2 macrophages we previously observed in murine sarcoma induced by 3‐methylcholanthrene following PPI treatment.^8^ Notably, EIPA/Cis and ESO treatments are also associated to a rise in the number of DCs in 4T1 and TS/A tumors, respectively. CD8^+^ T cells are also significantly increased by EIPA/Cis in 4T1, whereas ESO treatment only slightly increased CD8^+^ T cells in TS/A tumors. Inhibiting suppressive myeloid populations may restore antitumor CD8^+^ T‐cell responses.^43^ Along this line, a concomitant immune response against the tumor may be triggered by the treatments used in these studies, possibly fostered by the normalization of extracellular pH. Thus, the relevant surface expression of the two proton exchangers on different tumors may represent predictive markers of response or resistance to PPI or EIPA, used as anticancer drugs.

Amiloride and esomeprazole are clinically approved and largely used without causing relevant side effects. In the present study, mice displayed no detectable adverse reaction although treated with doses of ESO 2–5 times higher than the maximal safe dose used in human studies.^44^, ^45^, ^46^ Also, amiloride can be used at very high levels in humans without toxicity.^47^, ^48^ Clinical data on EIPA are still missing. However, the tolerability of EIPA is high in mice, as confirmed by Maidorn et al. that used high doses of the drug,^29^ suggesting that the chemical modifications present in EIPA do not increase the risk of adverse effects and that its translation to a clinical use is possible.

Chemotherapy is the only option for TNBC.^2^, ^3^ However, it is toxic and its beneficial effects are rapidly overcome by the development of resistance. The prolonged time line and the high cost of new drug discovery and development represent a limit for the generation of therapies with high efficacy on more malignant cancers.^49^ Our present study indicates that therapeutic combination regimens with nontoxic drugs approved for different therapeutic uses, which target tumor‐specific mechanisms, may result more efficient and safe than chemotherapeutics and may prevent the evolution of drug resistance. The combo therapy EIPA/Cis may represent a novel therapeutic approach ready, safe, cheap, and hopefully very effective on solid tumors which are resistant to classical therapies such as TNBC. Furthermore, the data obtained on the hormone sensitive TS/A tumor indicate that a single nontoxic drug, such as ESO, may provide in some tumors better results than chemotherapic drugs, without toxic effect. Therefore, drugs repurposing, with testing of known drugs for their efficacy in other diseases, such as cancer, may rapidly build a bond between research and clinic and provide low cost, safe drugs with high therapeutic efficacy.^50^

## ETHICS STATEMENT

All the animal experiments were performed under the Institutional Animal Care and Use Committee and were cared for in accordance with national legislative provisions for the protection of animals used for scientific purposes.

## CONFLICT OF INTEREST

The authors declare no competing interest.

## Supporting information

Fig S1

Fig S2

Fig S3

## Data Availability

The data that support the findings of this study are available from the corresponding author upon reasonable request.
