# Supplementary figures and images for: Therapeutic efficacy of proton transport inhibitors alone or in combination with cisplatin in triple negative and hormone sensitive breast cancer models
Source: Cancer Med. 2021 Nov 18;11(1):183–93. doi: 10.1002/cam4.4371 (PMC8704177; doi:10.1002/cam4.4371)

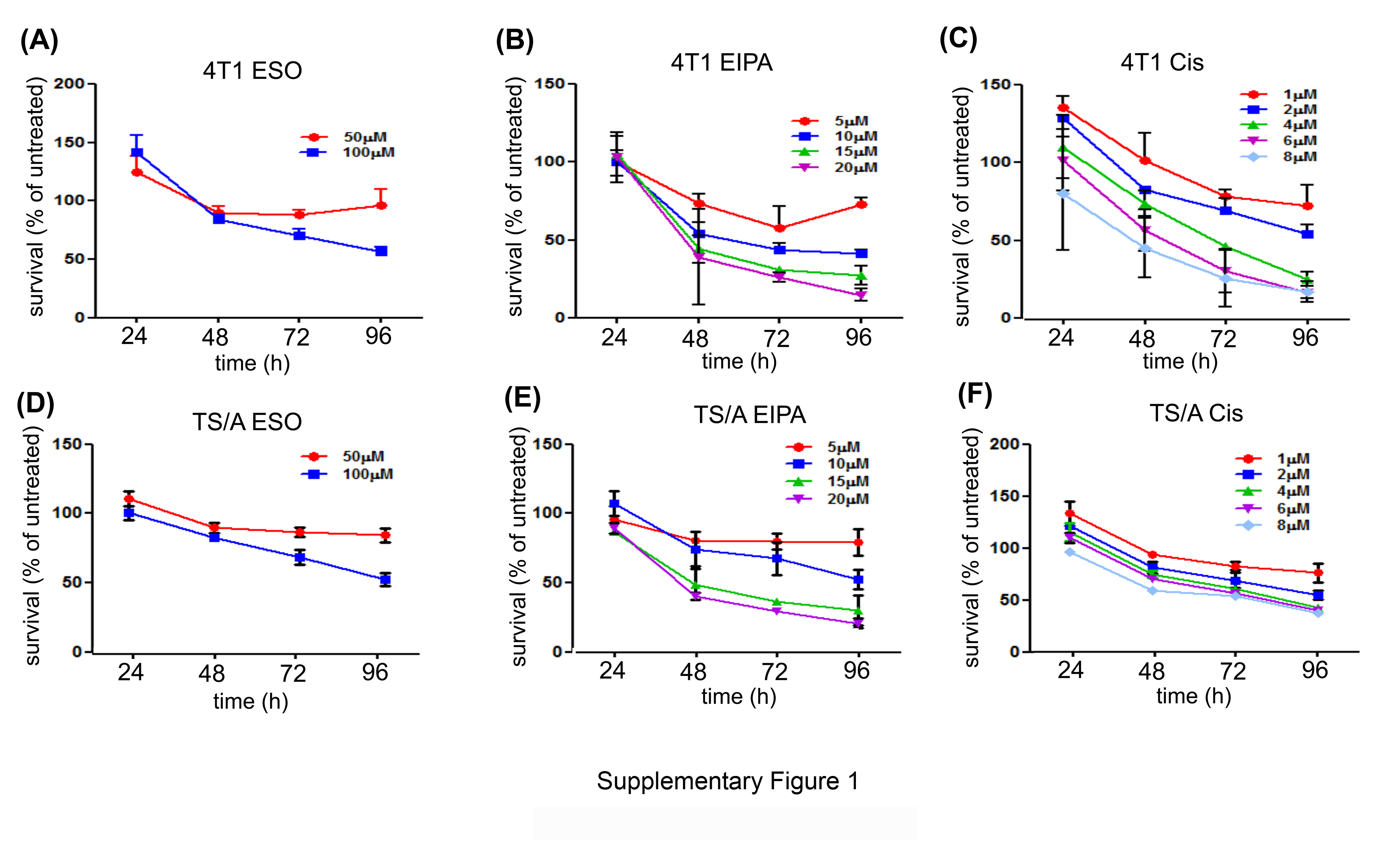

Supplement: Supplementary file 1 — Fig S1 [file CAM4-11-183-s001.tif]

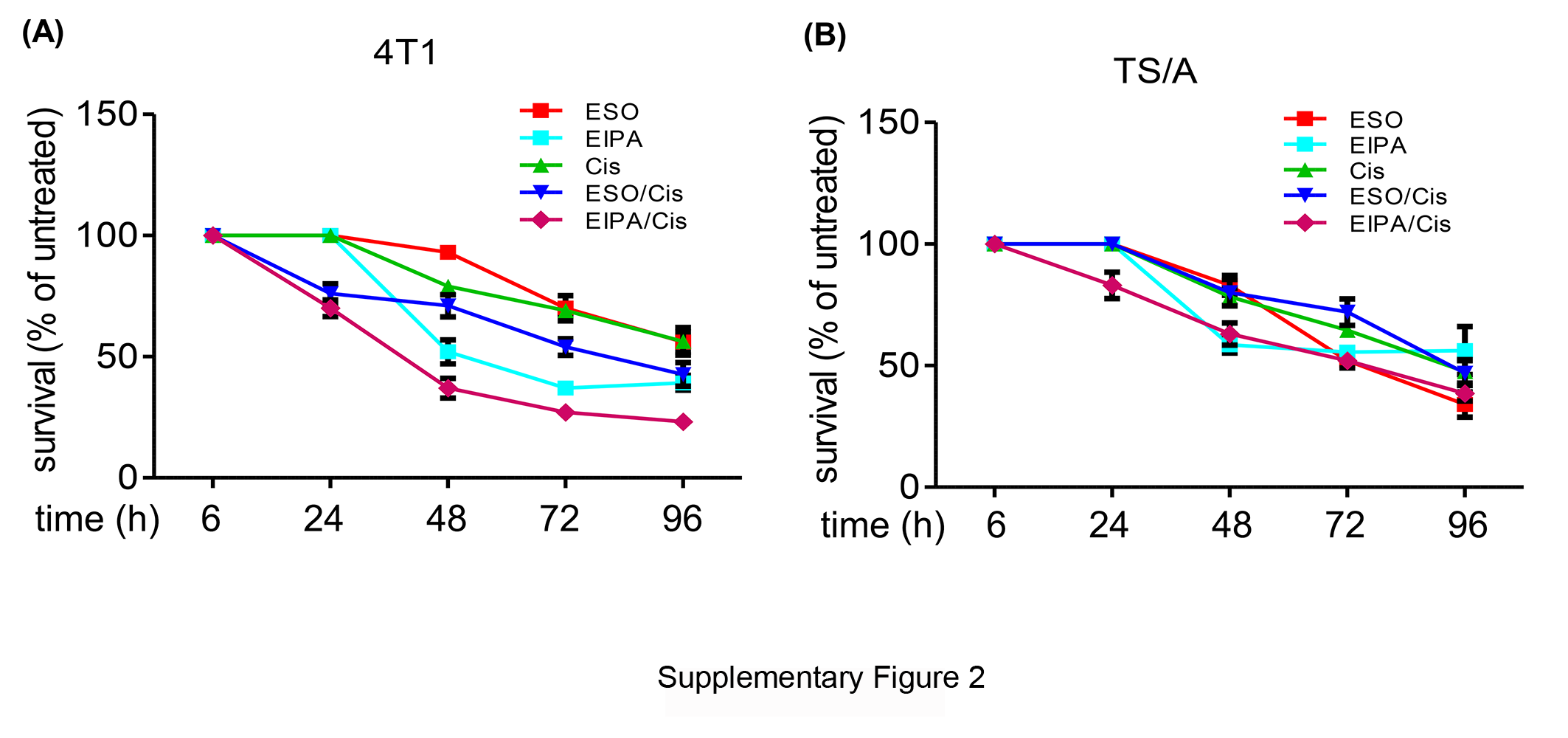

Supplement: Supplementary file 2 — Fig S2 [file CAM4-11-183-s003.tif]

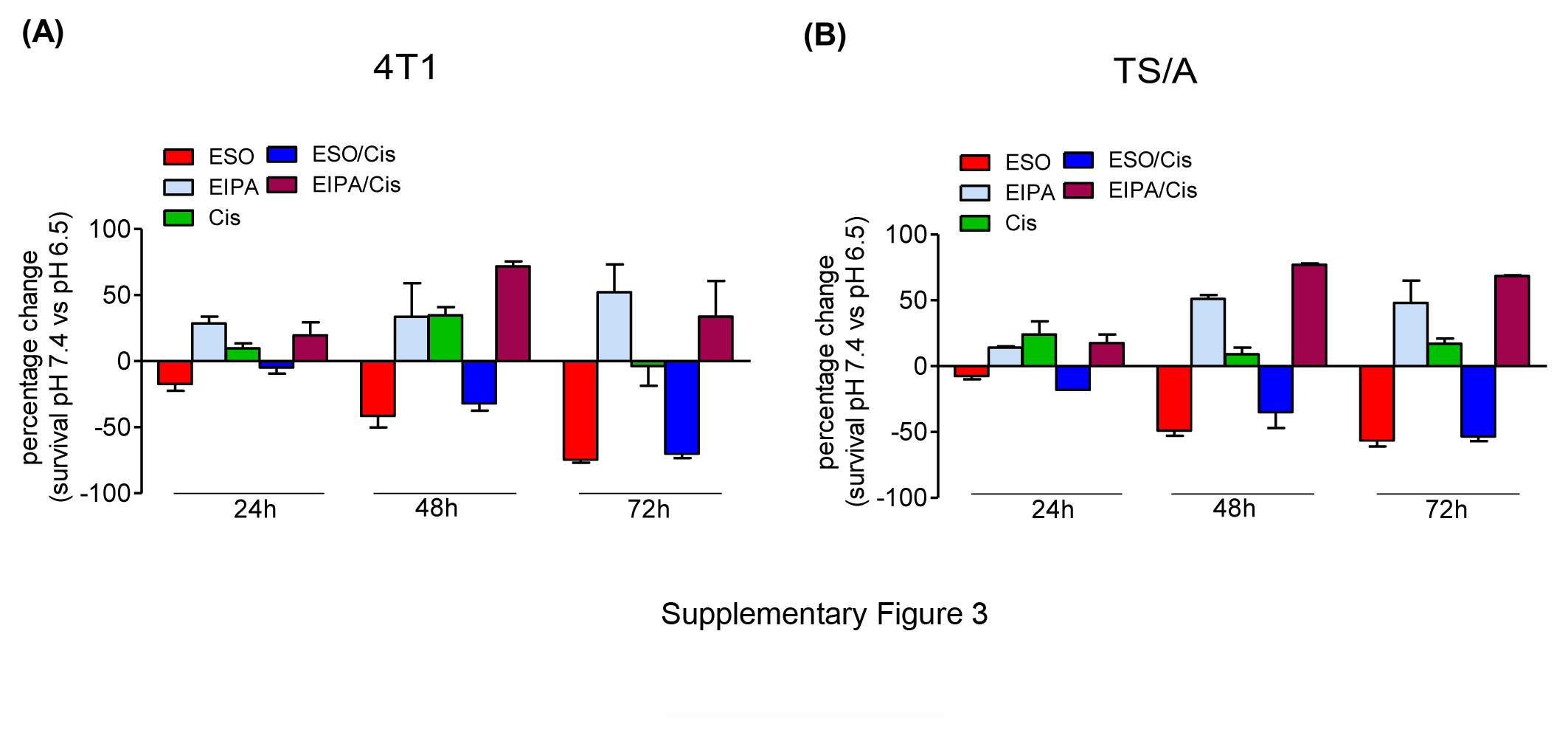

Supplement: Supplementary file 3 — Fig S3 [file CAM4-11-183-s002.tif]
